# Olfactory-colour crossmodal correspondences in art, science, and design

**DOI:** 10.1186/s41235-020-00246-1

**Published:** 2020-10-28

**Authors:** Charles Spence

**Affiliations:** Department of Experimental Psychology, Anna Watts Building, University of Oxford, Oxford, OX2 6GG UK

**Keywords:** Crossmodal correspondences, Odour-colour associations, Synaesthesia

## Abstract

The last few years have seen a rapid growth of interest amongst researchers in the crossmodal correspondences. One of the correspondences that has long intrigued artists is the putative association between colours and odours. While traditionally conceptualised in terms of synaesthesia, over the last quarter century or so, at least 20 published peer-reviewed articles have assessed the consistent, and non-random, nature of the colours that people intuitively associate with specific (both familiar and unfamiliar) odours in a non-food context. Having demonstrated such consistent mappings amongst the general (i.e. non-synaesthetic) population, researchers have now started to investigate whether they are shared cross-culturally, and to document their developmental acquisition. Over the years, several different explanations have been put forward by researchers for the existence of crossmodal correspondences, including the statistical, semantic, structural, and emotional-mediation accounts. While several of these approaches would appear to have some explanatory validity as far as the odour-colour correspondences are concerned, contemporary researchers have focussed on learned associations as the dominant explanatory framework. The nature of the colour-odour associations that have been reported to date appear to depend on the familiarity of the odour and the ease of source naming, and hence the kind of association/representation that is accessed. While the bidirectionality of odour-colour correspondences has not yet been rigorously assessed, many designers are nevertheless already starting to build on odour-colour crossmodal correspondences in their packaging/labelling/branding work.

## Significance Statement

Crossmodal correspondences – defined as the sometimes surprising associations that people experience between seemingly unrelated features, attributes, or dimensions of experience in different sensory modalities – constitute an important aspect of our everyday experience. Crossmodal correspondences between colours and odours have long been of interest to artists and, more recently, to designers wishing to convey the olfactory attributes of their products by means of colours (and colour schemes). However, progress in understanding, and, thus, incorporating, crossmodal correspondences into artistic and design practice have long been limited by the assumption that they constitute a form of synaesthesia. Over the last century, many academic studies have assessed the colours that people intuitively associate with a wide range of both familiar and unfamiliar odours. People tend to pick the colours that they do in response to specific odours because of the existence of statistically, structurally, semantically/lexically, and/or emotionally mediated correspondences. While the majority of psychologists have focussed their research efforts on assessing the colours that people intuitively associate with odours, designers are often more interested in the reverse correspondence, namely whether they can convey anything of the olfactory properties of a product by means of the use of well-chosen colours or colour schemes (i.e. combinations of colours). However, the fact that people typically refer to odours in terms of their source objects (as when they say ‘it smells like an orange’) and often struggle to name even those odours they are familiar with (at least when the latter are presented out of context), means that the bidirectionality of this class of crossmodal correspondence should not be assumed.

## Introduction

Interest in the study of the crossmodal correspondences has grown steadily in recent decades (see Spence, 2011, for a review). One of the correspondences to have caught the interest of painters and other artists is the association between colours and odours. While traditionally conceptualised in terms of synaesthesia (e.g. Kandinsky, 1977; Marks, 1978), more than 20 published peer-reviewed studies have now assessed the consistency (i.e. the non-random nature) of the colours (primarily hues, though, on occasion, also lightness/saturation) that people tend to associate with specific odours in a non-food context. Having demonstrated such consistent mappings in the general (i.e. non-synaesthetic) population (e.g. Demattè, Sanabria, & Spence, 2006; Gilbert, Martin, & Kemp, 1996), a number of researchers have recently extended this line of empirical research to ascertain whether such crossmodal associations are also shared cross-culturally (e.g. de Valk, Wnuk, Huisman, & Majid, 2017; Jacquot, Velasco, Spence, & Maric, 2016; Levitan et al., 2014; Nehmé, Barbar, Maric, & Jacquot, 2016), and to track their developmental trajectory (Goubet, Durand, Schaal, & McCall, 2018). There is growing interest in the existence of colour-odour correspondences from those in the design and marketing communities wanting to align their products’ olfactory attributes (e.g. in the case of perfume and food/beverage packaging/branding) with their visual communications.

Generally speaking, several different classes of explanation, including the statistical, semantic, structural, and emotional-mediation have been put forward to account for the existence of crossmodal correspondences (see Spence, 2011, 2018a, for reviews). A number of these explanations would appear to have at least some explanatory validity as far as justifying the existence, and nature, of the odour-colour correspondences that have been documented to date are concerned (Kemp & Gilbert, 1997; Schifferstein & Tanudjaja, 2004; von Hornbostel, 1931). However, before reviewing the empirical research on odour-colour crossmodal correspondences conducted over the last half century or so, I first want to draw attention to the early interest in the link between this particular pair of senses. The possibility that cross-sensory impressions might be elicited in those viewing colour as in a painting famously intrigued the likes of French post-Impressionist painter Paul Cézanne (1839–1906; Merleau-Ponty, 1964). Meanwhile, putatively synaesthetic connections between colour and odour were subsequently, albeit ultimately unsuccessfully, explored by Italian Futurist artists such as Azari (1924; described in Verbeek, 2017). There are also reports of symbolist performances at the end of the nineteenth century presenting corresponding colours, sounds, and scents to audiences (see Fleischer, 2007; Shepherd-Barr, 1999). Meanwhile, the famous Russian composer Scriabin often talked about, though never managed to deliver in his own lifetime, fragrances synchronised with the lighting score that he had designed for his 1911 tone poem *Prometheus: Poem of Fire* (Runciman, 1915). These days too, the exact nature of the link(s) connecting odours with colours continues to intrigue the international press (Beck, 2014), not to mention those in the design/marketing community (Kim, 2008, 2013; Schifferstein & Howell, 2015).

### Outline of the review

This narrative review traces the scientific findings that are most relevant to the crossmodal correspondences that have been documented between colour and odour. In the next section, the research on semantically mediated crossmodal associations, including those involving odour and flavour are summarised. At the same time, however, the common problem experienced by the majority of people as far as correctly naming/identifying odours when presented in the absence of any other contextual cues is also highlighted. In the third section, the literature on colour-odour correspondences in the world of flavour (i.e. food and drink) is summarised briefly. Next, the four major classes of explanation for crossmodal correspondences are outlined. Thereafter, in the following section, the scientific evidence concerning the odour-colour correspondences that have been documented in a non-food context is critically reviewed. In the penultimate section, I highlight the growing commercial interest that has emerged in the world of olfactory-visual crossmodal correspondences (Favre, 1968; Favre & November, 1979; Heatherly, Dein, Munafo, & Luckett, 2019; Kim, 2008, 2013; Lunardo & Livat, 2016; Schifferstein & Howell, 2015). This recent wave of applied interest primarily revolves around the optimisation (in terms of maximising processing fluency) of marketing communications, by helping designers set the most appropriate odour expectations by means of their use of colour cues (see Piqueras-Fiszman & Spence, 2015, for a review). The review ends by concluding that the study of ‘scented colours’ represents an intriguing example of the application of cognitive research to the field of design, and hence is a highly relevant topic for this journal.

## Crossmodal semantic associations between odours and colours

According to Nehmé et al. (2016, p. 34): *'Whereas the relationship between audition and vision has been well documented, a cross-modal linkage between the olfactory and visual senses is seldom mentioned in the literature’.* Contrary to this suggestion, however, I would like to argue that there have actually been many hundreds of scientific studies documenting crossmodal links between olfactory and visual stimuli. For instance, think only of the many studies that have been published investigating the effect of food colouring on flavour perception (see Spence, Levitan, Shankar, & Zampini, 2010, for a review). Given that this large body of food-related research has repeatedly been reviewed elsewhere, only the key insights and observations will be briefly summarised here. Meanwhile, the 20 or so published studies that have been conducted outside the world of multisensory flavour perception that are relevant to the nature of odour-colour crossmodal correspondences are discussed and critically evaluated. So, while it may well be the case that the total number of studies on olfactory-visual interactions pales in comparison to the multitude of audiovisual studies that have been published to date, there is nevertheless still a substantial body of research out there on colour-orthonasal odour correspondences. What is more, this disparate literature (in a non-food context) has not been brought together and critically reviewed previously.

The most popular explanation for the existence of odour-colour crossmodal correspondences in the general (i.e. non-synaesthetic) population is in terms of multisensory semantic object representations. The explanation for many crossmodal associations, such as the fact that a barking/woofing sound and the picture of a dog, or a meowing sound and the picture of a cat belong together is in terms of associative learning (e.g. Barros, Eppe, Parisi, Liu, & Wermter, 2019; Chen & Spence, 2017; Fifer, Barutchu, Shivdasani, & Crewther, 2013; Walker-Andrews, 1994). It should come as little surprise, therefore, to find that according to Stevenson, Rich, and Russell (2012), the primary means of crossmodal association between odours and colours occurs when an odour evokes a specific object (or presumably context/situation/environment) and a semantic match with an associated colour is made (see also Ward, 2014; Zellner, 2013). Goubet et al. (2018) provided indirect support for such a semantic account when they demonstrated that the better (i.e. more accurate) that people are at identifying the source of an odorant, the more accurate (or at least consensual) their colour matches tend to be. So, for example, 75% of those who correctly identified the odour of grenadine in Goubet et al.'s study picked red, whereas those who were unable to identify the source of the odour chose yellow, brown, and orange instead (cf. de Valk et al., 2017, for a similar result amongst a more linguistically diverse group of participants).

Stevenson et al. (2012) note that under those conditions where nothing specific comes to mind (i.e. when an odorant is neither nameable nor familiar), one may find that people’s colour matches are modulated by (or rather based on) the intensity, irritancy, and/or hedonics of the olfactory stimuli instead. The latter, then, suggesting a rather more direct perceptual match between disparate sensations (see also Jacquot et al., 2016; Nehmé et al., 2016, on this distinction). Though, as Nehmé et al. (2016, p. 34) point out, one might want to question whether the hedonic response is related more to semantics or perception in this case. However, as well as any object-specific associations that may be triggered by particular olfactory cues, the latter can also trigger memories that are themselves emotionally valenced. Think here only of the Proustian moment (Runciman, 1915; Van Campen, 2014). Such emotional associations with odours (no matter whether or not there is a specific memory attached) likely provide a basis for certain crossmodal correspondences between olfactory stimuli and colours.

### On the difficulty of identifying the source of an odour

One key difference between olfaction and the other senses is that we typically find it much harder to identify olfactory cues in the absence of any other contextual information than we do to identify visual cues (and object-related sounds, for that matter) when they happen to be presented in isolation; or at least that would seem to be the case for those visual and auditory stimuli that are typically used in laboratory research, which are normally rapidly identified. It is precisely this difficulty that people experience when they try to identify, or put a name to, an odour (when presented without any other identifying or contextual information; e.g. Cain, 1974; Yeshurun & Sobel, 2010) that perhaps explains what makes the study of odour-colour matching so interesting and, in a sense, unique. One might think of olfactory cues as providing a kind of semantic information, but one where the sensory input does not necessarily trigger the associated conceptual representation automatically in quite the same way that the sight or sound of an object, or action, would.

Crossmodal correspondences between odour and colour are, therefore, surprising inasmuch as they do not reference a specific source object (or at least not one that the participant is necessarily aware of). No one, I presume, would be in the least bit surprised that we associate the sight of a dog with a barking sound, nor, one presumes, with the smell of a dog either. These kinds of semantic, or categorical, crossmodal associations are undoubtedly also of interest to psychologists, but they tend to be studied separately from the crossmodal correspondences (see Chen & Spence, 2010, 2011, 2018a, b, on audiovisual semantic interactions).

What is also worth noting here is that whenever we succeed in identifying an odour, the typical response is to say that it ‘smells like’ the source object as when we describe a citrus aroma as ‘smelling like an orange’. This might lead one to think that crossmodal colour-odour associations would primarily be object-based, or semantic (this is what Majid & Burenhult, 2014, refer to as source-based descriptions). One of the things that is especially interesting about the sense of smell, though, is how poor we tend to be as far as identifying the source of olfactory stimuli in the absence of any other contextual information. The frequently mentioned situation where an odour is recognised as familiar, while its source object eludes us, is known as the ‘tip-of-the-nose’ phenomenon (Djordjevic, Zatorre, Petrides, & Jones-Gotman, 2004; Lawless & Engen, 1977).

Often, the olfactory stimuli that have been presented in laboratory-based studies have been linked to specific objects, situations, or environments (e.g. Gottfried & Dolan, 2003; Seigneuric, Durand, Jiang, Baudouin, & Schaal, 2010; Zellner, 2013). Under everyday conditions, however, there are normally a number of other (often visual) cues that typically aid in our ability to identify what it is that we are smelling. Even for those odours where no obvious source object or association comes to mind, people still often associate colours in a significantly non-random manner. In order to try and determine whether semantic and/or perceptual mechanisms are involved in driving such odour-colour correspondences, Stevenson et al. (2012) assessed the odour characteristics (e.g. nameability, familiarity, intensity, irritancy, hedonics) that co-varied with the crossmodal matches.

On occasion, some unusual (i.e. less intuitive) crossmodal associations have also been noted. Examples here include everything from the smell of vinegar being matched with the colour pink in a study by Stevenson et al. (2012), through to the smell of mushroom being matched with the colours blue/yellow in a study by Spector and Maurer (2012). Such unexpected odour-colour matches are, though, pretty rare. What is more, they might well reflect the colour of the branding in which these products are normally experienced, if not the source object of the odour itself. However, even for those olfactory stimuli that are associated with a concrete source object such as, for example, an apple, there can still be multiple ‘correct’ colour matches. One might, for instance, legitimately associate the smell of apple with the white flesh of the fruit itself, the muddy golden-yellows of apple juice, the red, green, or pink colour of the skin, or even the branded colours of the packaging associated with the farm of supermarket that typically happens to provide your fruit at home (cf. Mankin & Simner, 2017).

While people often struggle to identify the source object associated with an odour in the absence of any other contextual information, they can often say whether or not it is familiar. They are also likely able to make an edibility judgment and a hedonic assessment as well. Neuroimaging studies have revealed the recruitment of the primary visual cortex and the visual association cortex suggesting that people may well try to mentally imagine visually the source of the odour that they happen to be smelling (Qureshy et al., 2000; Royet et al., 1999). The role of the visual cortex in olfactory naming/identification perhaps providing a part of the explanation for why it is that what we see typically has such a dramatic effect over what we smell. According to Royet et al., judging comestibility, rather than familiarity, led to increased activation in visual areas 17–19 (though it is worth noting that these judgments were highly correlated). Meanwhile, Qureshy et al. found that the cuneus (areas 18, 19), the cingulate gyrus, the insula, and the cerebellum were all involved when the participants in their study tried to put a name to the various familiar olfactory stimuli that they were presented with.

The crossmodal influence of colour on orthonasal odour identification/discrimination performance has been documented in a large number of studies (e.g. Blackwell, 1995; Davis, 1981; Demattè et al., 2006; Demattè, Sanabria, & Spence, 2009; Stevenson & Oaten, 2008; Zellner, Bartoli, & Eckard, 1991; see also Ferrier et al., 2009; Hörberg et al., 2020). However, by far the most extensively studied of crossmodal interactions between olfactory and visual stimuli happens to be in the area of multisensory flavour perception. It is worth noting that these studies have had participants evaluate and rate odorants in flavoured food and drink that have been experienced by either the orthonasal, or retronasal routes (Spence, 2015a).

## Colour-aroma correspondences in the context of multisensory flavour perception

One strand of the research that is relevant to the topic of colour-odour correspondences concerns the colours that we associate with the flavours of everyday food and drink products. It is worth stressing here that this body of sensory science research stands somewhat apart from the more abstract crossmodal mapping of colour and odour where there is not an obvious common source object to explain the crossmodal connection. The research in this area shows that people typically associate flavours (i.e. retronasally experienced aromas when combined with gustatory stimulation) with colours and, on occasion, colours with flavours too (Wan et al., 2014). Separately, it has also been shown that changing the colour of a food or, more often, drink typically alters the taste/flavour as well (see Spence, 2015d). In the majority of cases, the colour and odour/flavour are typically presented in the same substrate (or source object). In such studies, though, it is normally clear that both colour and odour relate to the same food/flavour object (and that they are likely to be both edible and hedonically positive). The fact that both colour and odour, no matter whether the latter is experienced orthonasally or retronasally, are linked to the same flavour object presumably means that the participant will assume that the individual sensory cues are semantically related.

Over the years, a large number of studies have investigated crossmodal correspondences between the colour and flavour/aroma of food and drink (e.g. Arao, Suzuki, Katayama, & Yagi, 2012; DuBose, Cardello, & Maller, 1980; Oram et al., 1995; Parr, White, & Heatherbell, 2003; Petit, Hollowood, Wulfert, & Hort, 2007; Wang & Spence, 2019; Zellner & Durlach, 2003; Zellner & Whitten, 1999). For instance, the evidence from my own laboratory has shown that colours are associated with specific flavours (which are sometimes different for different individuals), and that these flavour/aroma expectations can then bias the reported flavour/aroma, at least if the degree of discrepancy between the colour (and the odour/flavour expectations that that colour gives rise to) and the actual odour is not too great (see Shankar, Levitan, et al., 2010; Shankar, Simons, Levitan, et al., 2010; Shankar, Simons, Shiv, Levitan, et al., 2010; Shankar, Simons, Shiv, McClure, et al., 2010).

The colour of the packaging in which food and drink products are so often presented represents another level at which colour-odour correspondences may operate (see Spence & Velasco, 2018, for a review). Many people, after all, associate the taste/flavour of a cola drink with the red and white colour scheme of Coca-Cola rather than necessarily the brown colour of the drink itself. In this case, however, while packaging colour is sometimes closely linked to a specific flavour/aroma in a food or beverage product that is certainly not always the case (Zellner et al., 2018). Zellner and her colleagues conducted an intriguing study showing that sweetie wrappers (which come in all manner of colours unconnected to the taste/flavour of the confection inside) did not affect taste/flavour ratings. Hence, the crossmodal associations between, not to mention the crossmodal influence of, packaging colour and product aroma/flavour is likely to be product dependent.

The explanation for such crossmodal correspondences between colour of product or distinctive packaging, and aroma/flavour judgments is almost exclusively in terms of associative learning. That said, the way in which food companies choose to colour their food and beverage products might itself be expected to be based on both the natural statistics of the environment and also possibly a fundamental intensity-based correspondence such that more intensely flavoured products are normally more intensely saturated in colour. At the same time, however, novel colour-aroma/flavour associations are sometimes introduced into the marketplace, as evidenced, for instance, by the growing popularity of blue drinks (see Shankar, Levitan, & Spence, 2010; Spence, 2018b).

While the focus of this review, and in the literature more generally, has been on crossmodal correspondences with orthonasally experienced odours (i.e. as when sniffing), Koza, Cilmi, Dolese, and Zellner (2005) highlighted qualitatively different patterns of crossmodal influence of colour depending on whether participants experienced odorants orthonasally or retronasally (the latter experienced when eating and drinking). In particular, they reported that while colour extered a significant influence over reported orthonasal aroma judgments (as when sniffing) they had no effect on retronasal aroma judgments when the coloured flavoured solution was already in the mouth. Their post-hoc explanation for this unexpected result, one of the few reported crossmodal differences between orthonasal and retronasal smell, was that it may be especially important to be able to correctly judge the taste/flavour of those foods that one is about to swallow, hence meaning that certain crossmodal interactions are less commonly observed.

## Explaining the correspondences

Over the years, several distinct classes of explanation have been put forward to try and account for the wide range of crossmodal correspondences that have been documented to date (see Spence, 2011, 2018a, for reviews). It is worth noting that such explanations are normally only invoked under those conditions in which the various unisensory cues are not tied to a common source object, as for the coloured foods and drinks reviewed in the previous section, and where explanations have been framed in terms of crossmodal semantic congruency instead (Chen & Spence, 2011, 2018a, b). Perhaps the most powerful explanation, in terms of being able to account for the largest number of the crossmodal correspondences, is in terms of the internalisation of the statistics of the environment (see Parise, Knorre, & Ernst, 2014; Spence, 2011). The research shows that novel correspondences, at least when envisaged as coupling priors in terms of Bayesian causal inference (see Chen & Spence, 2017, for a review), can be acquired/internalised with surprisingly little exposure to an arbitrary new mapping of stimuli, or stimulus dimensions.

For instance, in the case of the association of basic taste properties with olfactory stimuli, this has been shown to occur in a matter of trials, and even in the absence of awareness (see Stevenson & Boakes, 2004, for a review). Meanwhile, Ernst (2007) established a novel crossmodal correspondence between the dimensions of haptic stiffness and visual brightness with little more than an hour or two of training. In the case of those crossmodal correspondences between odour and colour, it is certainly possible that odour intensity and colour saturation might well be correlated in terms of the natural statistics of the environment. On this point, one need only think about the intensely flavoured and coloured processed food and drink products that one finds in the supermarket. That said, the correlation between colour and odour may have been more pronounced in previous centuries – that is, prior to the development of synthetic chemistry. The latter allowed for a more complete dissociation between these senses than had been the case formerly, when most colouring agents, be it for clothing or food, would have had their own distinctive smell (see Blaszczyk & Spiekermann, 2017; Moore, 2016; Woolgar, 2018).

A second class of explanation for the existence of crossmodal correspondences is in terms of emotional mediation (Schifferstein & Tanudjaja, 2004). The basic idea here is that we may feel that certain stimuli belong together because individually the unisensory stimuli are either associated with, or else evoke, the same, or similar, emotion (see Palmer, Schloss, Xu, & Prado-León, 2013). Both colours (e.g. Gilbert, Fridlund, & Lucchina, 2016) and olfactory stimuli (e.g. Benderly, 1988; Hinton & Henley, 1993; Schiffman, 1974) tend to be robustly associated with emotions (see also Arao, Suzuki, & Yagi, 2011). As we will see below, the emotional mediation account provides a plausible explanation for a number of the odour-colour correspondences that have been documented to date in the case of those odorants that are unfamiliar, and hence unlikely to be linked with a specific source object (e.g. food or drink).

A third class of correspondence that has been suggested is a structural correspondence based on a shared neural representation used by different stimulus modalities to code, for example, intensity, or magnitude. According to Stevens (1957), increasing stimulus intensity is associated with increased neural firing in every sense. This commonality at the level of neural coding offers a potential basis for matching sensations. As we will see below, several researchers have indeed demonstrated a correspondences between perceived odour intensity and visual brightness/lightness (Kemp & Gilbert, 1997; von Hornbostel, 1931), though whether any such correspondence reflects a direct crossmodal mapping of stimulus brightness, or intensity, or is instead simply inferred, has long been debated (see Cohen, 1934; Hartshorne, 1934).

According to the lexical/semantic account of crossmodal correspondences, sensory qualities might come to be associated because of the use of common lexical terms used to describe them, as when one talks of a 'sharp' cheese, or a ‘loud’ pair of bright red trousers (Spence, 2018a). The semantic coding account of the crossmodal correspondences, originally suggested by Martino and Marks (1999, 2000), was first evidenced in speeded classification tasks. According to Walker, Walker, and Francis (2012), though, 'lexical' may be a more appropriate term than 'linguistic' in this context. However, regardless of the preferred terminology, the use of a common term to describe different sensory attributes may ultimately build on the statistical correspondences mentioned above, as when so many languages/cultures use the terms high and low when referring to sounds of differing pitch (see Parkinson, Kohler, Sievers, & Wheatley, 2012). Walker et al. (2012) extended the semantic account by suggesting that crossmodal correspondences between auditory, visual, and tactile stimuli may be based on their shared connotative meaning (as assessed, for example, by means of the semantic differential technique; see also Walker, 2012).

Rather than seeing these four classes of explanation as being mutually exclusive, the suggestion that has emerged from the literature over the years is that different combinations of these explanations may be most appropriate when trying to account for different kinds of crossmodal correspondence (see Spence, 2011, 2018a, for reviews). As we will see below, all four accounts help to explain at least certain of the findings from the more than 20 studies that have been published to date on the topic of crossmodal correspondences between olfaction and colour (in a non-food context).

## Assessing the evidence for odour-colour crossmodal correspondences

I now wish to summarise the evidence from those experimental psychology/psychophysics studies that are relevant to the nature/existence of odour-colour crossmodal correspondences. As will soon become clear, a growing body of research has attempted to extend the findings from WEIRDo’s – this, the acronym introduced by Henrich, Heine, and Norenzayan (2010), to draw attention to the fact that the vast majority of participants in psychology research tend to be drawn from a very narrow demographic of those living in Western, Educated, Industrialised, Rich, and Democratic societies. Very often, in fact, they happen to be psychology students at university in North American (see Arnett, 2008). In recent years, there has been growing interest in extending the study of colour-odour crossmodal correspondences both across different cultures and, to a lesser extent, across the age spectrum too. Table 1 summarises the various studies of odour-colour crossmodal correspondences that have been published to date.

**Table 1:** Summary of peer-reviewed published studies that have demonstrated a consistent (i.e. non-random) crossmodal mapping of colours to odours (or vice versa; see Langner, 1997). While the number and type of olfactory stimuli, the number and type of visual stimuli, and the participant demographics have varied widely between studies, all have demonstrated the existence of crossmodal correspondence between colours and odours. The precise nature of the mapping, though, often varies from one study/participant sample to another. Note that certain studies have chosen to match olfactory stimulus intensity, whereas others have deliberately chosen to vary perceived olfactory intensity. The various studies listed in the table also vary in terms of whether the olfactory stimuli were easily associated with a source object or not. WEIRD is the acronym used to refer to Western, Educated, Industrialized, Rich, and Democratic undergraduate participants (very often psychology students) who constitute the subject pool for the majority of experimental psychology research (e.g. Arnett, 2008; Henrich et al., 2010)

Study	Number and type (in brackets) of:			Participant	X-modal mapping		
	Participants	Colours	Odours	group	LI	CM	SM
Von Hornbostel (1931)	3 or 4	Greyscale	c. 800	WEIRD	X?		
Déribéré (1978)	1000	Free written response	21 Od names	General public		X	X
Fiore (1993)	89 (US women)	11 Solid/19 Patterns	3 (Od/Fr)	WEIRD	X?		
Gilbert et al. (1996)	E1–94 / E2–50 (US)	E1 = 11 (words)/E2 = 1565 (colours)	20 (Ob/Od)	General public		X	X
Kemp and Gilbert (1997)	38 (US)	1565	5 × 3 (Od)	WEIRD	X		
Langner (1997)	c. 120 (Germany)	15–20 abstract photos	Free response	WEIRD		X	
Schifferstein and Tanudjaja (2004)	69 (NL Females)	96 (colours)	14 (Fr)	WEIRD	X	X	
Österbauer, Matthews et al. (2005; Österbauer, Sanabria, et al., 2005)	40 (UK)	10 (hues)	17 (Ob/Od)	WEIRD		X	X
Demattè et al. (2006)	21 (UK)	10 (hues)	6 (Ob)	WEIRD		X	X
Zellner, McGarry, Mattern-McClory, and Abreu (2008)	E1–4: 63/52/60/68 (US)	11 (colours)	6 (Fr)	WEIRD		X	
Spector and Maurer (2012)	78 (Canada)	Free verbal response	22 (Ob)	WEIRD		X	X
Stevenson et al. (2012)	18 (Australia)	Pick colour on computer	20 (Ob)	WEIRD		X	X
Kim (2013)	70 (Korea)	120 (colours)	8 (Fr)	WEIRD	X	X	
Maric and Jacquot (2013)	155 (France)	24 (colours)	16 (Ob)	WEIRD	X	X	X
Levitan et al. (2014)	122 (6 cultural groups)	36 (colours)	14 (Ob)	Cross-cultural		X	X
Schifferstein and Howell (2015)	66 (NL)/80 (US) Women	5 (colour schemes)	5 (Fr)	WEIRD		X	
Jacquot et al. (2016)	59(UK)/60(France)	24 (colours)	16 (Ob)	Cross-cultural		X	X
Nehmé et al. (2016)	155(France)/96(Lebanon)/110(Tw)	24 (colours)	16 (Ob)	Cross-cultural		X	X
Adams and Doucé (2017)	284 (Belgium)	Bright-dim/light-dark	32 (Ob/Fr)	WEIRD			X
De Valk et al. (2017)	11(Maniq)/24(Thai)/24(NL)	84 (colours)	15 (Ob)	Cross-cultural		X	X
Goubet et al. (2018)	186 (France)/337(US)	8 (hues)	8 (Ob)	Cross-cultural/development		X	X
Kaeppler (2018)	30 (Germany)	Free response	10 (Ob)	WEIRD		X	X
Heatherly et al. (2019)	E1–50/E2–52 (US)	4 (hues/wine labels)	5 (Ob)	General public		X	

*Od* odorants, *Fr* fragrance, *Ob* objects (typically edible), *LI* lightness-intensity; inverse relation between colour lightness and perceived odour intensity, *CM* consistent matching, *SM* source matching, meaning that the colour-odour link is mediated by specific object associations, *WEIRD* Western, Educated, Industrialised, Rich, and Democratic

The detailed summaries of the various studies provided in this section will hopefully help to make clear just how diverse the methods that have been used to date have been. Particularly salient differences between published studies include in terms of the familiarity (or otherwise) of the olfactory stimuli used, and the wide versus narrow range of colours that the participants in these various studies have had to choose from. The studies reviewed in this section also tend to split in terms of whether the odours used have more to do with perfumery or food/floral/herbal/object scents. What is also noticeable is how few of the studies have actually asked the participants concerned whether they find it easy or difficult to select matching colours for odours. Note that simply demonstrating a consensual match tells us nothing about how intuitive, or natural, participants find it to make such cross-sensory connections. A final point to be aware of here when reading the descriptions of the studies outlined is how the participants have nearly always been asked to pick a colour to match an odour, rather than vice versa. This contrasts with much of the design work discussed in the next section where the aim has typically been to go the other way, namely to pick colours, and develop colour schemes that people will match to the aroma or fragrance of the product contained within.

So, what exactly do those studies that have attempted to assess the mapping of colours to odours show? In what was perhaps the first study of its kind, von Hornbostel (1931) had his participants match odours to points on a grey scale and also to auditory pitch. The three or four participants who took part in this early study had to make pairwise judgments of smell-lightness (or smell-brightness ‘*Geruchshelligkeit*’) for approximately 800 pure undiluted chemicals. On the basis of the results, von Hornbostel was able to order the odours according to their position on the grey scale (i.e. in terms of increasing smell lightness). Intriguingly, he then attempted to link these judgments of olfactory brightness to the underlying chemical structure of the odorants involved (e.g. in terms of carbon-chain length, the number of double and triple bonds, the number of aromatic rings, etc.), though few further details are given in this early study.

Almost half a century later, Déribéré (1978) summarised the results of a large-scale survey of people’s colour choices in response to seven basic classes of odour, namely camphor, musk, floral, mint, ethereal, acrid, and putrid (cf. Amoore, 1970). Colour associations were also collected for a number of other named odours (amber, tobacco, musk, rose, jasmine, carnation, and verbena) though the results were not reported. Of the 3000 questionnaires sent out, around 1000 useable replies were received. Of these, only 100 were clearly intuitive and only ten showed a direct and well-characterised synaesthetic mapping, meaning the visualisation of a specific colour when a given odour was presented*.* In terms of the colour choices made by the remaining 900 non-synaesthetic individuals, Déribéré (1978, p. 116) concluded that certain well-defined colours were naturally associated with various commonly experienced odours*.* So, for example, white and light yellow were matched with camphor; red-brown or golden brown with musk; rose with floral; green with mint; ethereal with white or light blue; acrid with grey or maroon-brown, and dark-brown and black with putrid odours.

In a somewhat unconventional study, Fiore (1993) presented 89 female textile students with 30 fabric swatches varying in terms of their colour and patterning. The participants evaluated each swatch in terms of its visual and tactile qualities. They were then presented with three fragrances (White Shoulders, Samsara, and Coco, described as expressing predominantly floral, chypre, and oriental notes) and rated each odour-fabric combination in terms of the following statement ‘the person who wears *this* fragrance would like to wear this fabric’ along a 7-point scale. Agreement with the statement varied as a function of the material-odour pairing. Though the results are a little hard to decipher, they would at least appear to support the view that physical colour parameters, such as intensity and value contrast, differed between those combinations of colour and fabric that were rated high versus low on the question.

Gilbert et al. (1996) reported a study in which 94 participants were presented with 20 equi-intense odorants (selected from a range of fragrance materials commonly used in commercial perfumery): aldehyde C-16; bergamot oil, birch tar oil, caramel lactone, cinnamic aldehyde, civet artificial, 2-ethyl fenchol, galbanum oil, jasmine absolute, lavender oil, lilial, methyl anthranilate, methyl salicylate, neroli oil, olibanum oil, patchouli oil, pine oil, rosalva, star anise oil, and tarragon oil. The participants were given 11 possible colour terms: red, orange, yellow, green, blue, purple, pink, white, brown, grey, and black to choose from. They had to indicate the rating of fit for each odour by distributing 5 points across the 11 colour terms. Significant colour characterisations were documented for all of the odours. What is more, a subset of 15 participants who were brought back to the laboratory more than 2 years later showed a high degree of consistency in terms of their colour choices (the test-retest correlation was *r* = .53).

In a second experiment, Gilbert et al. had 50 new participants pick a colour from amongst 1565 colour chips varying in hue, saturation, and brightness. Regardless of the method used, consistent crossmodal mappings were obtained. In fact, 13 of the 20 odorants used were associated with a hue colour in the range of red-purple through green-yellow in a significantly non-random manner. Analysis of the data using circular statistics revealed that hue matches fell within a large range on the hue circle: red-purple, red, yellow-red, yellow, and green-yellow. No colour matches were found in the range of green, blue-green, purple-blue, blue, and purple. According to the study’s authors, though, it was unclear whether this reflected anything more than an artefact of the particular stimuli that had been chosen. In addition to differences in hue, the Munsell value and chroma also varied significantly as a function of the odour.

In a follow-up study, Kemp and Gilbert (1997) presented 38 participants with five odours at three concentrations each. The odorants – caramel lactone, cinnamic aldehyde, aldehyde C-16, galbanum oil, and methyl anthranilate – were chosen because of the strong and consistent colour associations in the earlier study by Gilbert et al. (1996). The 15 odour stimuli were presented in a random order with participants picking one of 1565 Munsell colour chips in order to match each odour. The odorants were then presented a second time and the participants rated the perceived intensity of each odorant. Kemp and Gilbert looked for any systematic variation in hue, chroma (degree of saturation), and value (colour brightness from white to black) linked to the variation in perceived odour intensity. The results revealed a dimensional relationship, such that colour lightness varied systematically (inversely) with perceived odour intensity for three of the five odorants (methyl anthranilate, cinnamic aldehyde, and galbanum). That is, a significant negative correlation was documented between Munsell value and perceived odour intensity, with darker colours being associated with the stronger odours.

According to Schifferstein and Tanudjaja (2004), even complex perfumes have corresponding colours, though they would appear to be largely the result of associations with the emotional dimension of pleasure. The participants rated the ‘degree-of-fit’ between 14 fine fragrances (11 female and three male: namely Aqua, Aromatonic, Beautiful, Boss, Calandre, DKNY, Escada, Indecence, Kouros, Le Male, Miss Dior, Opium, Paris, and Wish) and 96 colour samples. The participants also had to rate 15 emotional scales for the colour stimuli and separately for each of the olfactory stimuli. The participants undertook the following four tasks: odour-colour matching (the participants were also allowed to pick secondary and tertiary colours if they wanted), rating odours on the 15 emotion scales, rating the degree-of-fit of odour-colour pairs (including 17 colours) on a 9-point scale, and finally rating the 17 colours on the 15 emotion scales (with the various responses reduced down to the three main semantic differential dimensions of pleasure, arousal, and dominance). A consistent negative relationship was documented between odour-colour ‘degree-of-fit’ ratings and the magnitude of the difference between odours and colour scores on one of the emotion dimensions, namely pleasure. Consistent differences were also reported in terms of the colours that were associated with certain of the fragrances. For example, Wish was primarily associated with red and orange, Kouros with blue, and DKNY with yellow. Taken together, though, it was the blackness (brightness) dimension that showed the largest differences between fragrances, with less variance in terms of either chromaticness (saturation), or hue. In fact, variations in the latter two dimensions were mostly driven by responses to just one fragrance (Wish).

Österbauer, Matthews, Jenkinson, Beckmann, Hansen, and Calvert (2005) conducted a psychophysical odour-colour matching study followed by a functional magnetic resonance imaging (fMRI) neuroimaging experiment. In the psychophysical part of the study (see Österbauer, Sanabria, Calvert, Matthews, & Spence, 2005, for details), 17 odorants were presented, to the participants who had to pick the best-matching and worst-matching of ten approximately isoluminant colour patches (red, yellow, green, blue, orange, pink, brown, turquoise, purple, and grey). Galbanum, aldehyde C-16, cinnamic aldehyde, methyl anthranilate, and caramel furanone were all chosen because Gilbert et al. (1996) had previously reported that these odorants elicited strong crossmodal odour-colour associations. A further ten odorants (orange, strawberry, peppermint, grape, lemon, apple, banana, plum, spearmint, and cucumber) were essential oils of various fruits and herbs, extracted from natural sources. Their hypothesised colour associations were based on their naturally occurring colour (e.g. banana and yellow). One fragrance, ‘Out at sea’, was chosen as a control, without any prior hypothesis about a colour association, because no object or food item was associated with it. The final odorant, benzaldehyde, was chosen because it can be associated with both cherry and almond (i.e. red and yellowy-white, respectively).

The participants first selected the best-matching colour for each odorant, before indicating the certainty of the match on a scale from 0% (very unsure) to 100% (absolutely sure). Next they rated the odorant on a 7-point scale with respect to its pleasantness (very unpleasant to very pleasant), intensity (very weak to very strong), and familiarity (completely unknown to very familiar). The participants then wrote down the name of the odorant if they could identify it, before selecting a colour from the display which least matched the odour, followed by another certainty judgment. As predicted, the majority of the odours (all except aldehyde C-16, methyl anthranilate, cucumber, and ‘Out at sea’) showed significant associations with one or more of the colours. The majority of these odours were matched to the prototypical colour of the objects that they are naturally associated with (i.e. lemon-yellow). However, a number were matched to colours that one would not intuitively think that they were naturally associated with (e.g. pink was the second significantly paired colour with the aroma of banana after yellow). At the same time, none of the odorants were matched to the colours blue, purple, or grey (cf. Gilbert et al., 1996). On average, the participants were quite confident of their crossmodal matches.

A similar analysis revealed that for all but six of the odorants (galbanum, aldehyde C-16, cinnamic aldehyde, methyl anthranilate, caramel furanone, and benzaldehyde) there were colour associations that were significantly often chosen as matching least well. Intriguingly, grey was chosen as the least appropriate colour for most of the odorants with the exception of peppermint and spearmint that were matched to red and the ‘Out at sea’ odour, matched to red and brown. Interestingly, the average certainty for choices of the mismatching colour was significantly lower than for the colour-matching condition.

Demattè et al. (2006) assessed the consistency of the colour associations made by a group of 21 university students in response to six familiar odorants: caramel, cucumber, leather, lemon, spearmint, and strawberry. The olfactory stimuli were delivered by means of a computer-controlled olfactometer. The participants were instructed to pick the best-matching of ten simultaneously displayed colour patches (red, orange, yellow, green, turquoise, blue, purple, pink, brown, and grey). Each odorant was presented ten times. The results revealed one to three consistent (i.e. significantly non-random) colour selections for each of the six odorants. The synthetic aroma of strawberry was associated with the pink colour patch on 42% of trials and with red on 32% of occasions. Meanwhile, the spearmint odour was associated with turquoise (59%) – this the strongest association, while the cucumber odour was associated with the green colour (35% of trials). Though no attempt was made to assess whether the correspondence was based on a specific source object/stimulus match, this would seem like a reasonable possibility, given the nature of the olfactory stimuli chosen.

Zellner et al. (2008) conducted four experiments in which participants had 5 points to distribute across 11 colour terms to best describe a range of fragrances. In Experiment 1, there were three feminine fragrances (Beautiful, Calyx, and Tresor), and three masculine fragrances (Ralph Lauren Polo, Hugo Boss, and Drakkar Noir). The participants also rated the intensity, pleasantness, masculinity and femininity of the fragrances, and tried to identify them. The colour terms were: red, orange, yellow, green, blue, purple, brown, white pink, grey, and black. Significantly non-random mappings of colours were reported for all six fragrances. The ‘female’ fragrances tended to attract a lighter colour palate (pink, white, and yellow) than the fragrances that were perceived as male (where there were a predominance of green, blue, and brown responses). Experiments 2–4 presented two unisex fragrances (CKOne and CKBe). Those who judged the fragrances as masculine (or who were told that that was the case) picked darker colours (e.g. blue and to a lesser extent green) than those who considered the fragrances as feminine (e.g. predominantly picking pink and to a lesser extent yellow).

Spector and Maurer (2012) conducted a study with 78 participants involving 22 odorants: almond, anise, anisole chemical/-like smell, bergamot, camphene (camphor), cedar, cinnamon, eucalyptus, geranium, ginger, juniper berry, lavender, lemon, menthol, napthene (moth balls), mushroom, musty, onion, peppermint, rosewood, vanilla, and violet. The odorants varied in terms of their expected familiarity and pleasantness (varying between pleasant and unpleasant). The participants were instructed not to think of the source of the odour, and instead to verbally report any colour and texture associations (i.e. free association) that sniffing each odour brought to mind. The participants also rated the difficulty of the matching task, and the extent to which these kinds of associations ‘made sense’, on 7-point scales. A subset of the participants (*N* = 41) were subsequently brought back to try and identify the object associated with the smell. The participants’ verbal colour responses were then classified into one of 12 categories: red, orange, yellow, green, blue, purple, pink, white, brown, grey, black, and clear.

Significantly non-random colour choices were obtained for all 22 of the odorants. That said, 44% of participants reported associating colours with odours to be a difficult task, with only 36% reporting finding it easy. By contrast, 91% of participants reported associating texture to odours to be difficult. Nevertheless, at the same time, 65% of the participants agreed that the crossmodal matching task made intuitive sense. Spector and Maurer (2012) suggested that their results could perhaps be explained by a combination of source identification (lemon – yellow) for the four correctly identified odorants combined with natural biases (crossmodal associations) linking dimensions of experience for those odorants whose source was not correctly identified (*N* = 18 odorants).

Stevenson et al. (2012) had 18 participants evaluate 20 odours varying in terms of their semantic (i.e. familiarity and nameability) and perceptual (i.e. intensity, irritancy, and hedonics) attributes. The odorants included: almond essence, plastic, garlic, cut grass, strawberry, coal-tar soap, durian, caramel, vinegar, mushroom, pine, Brut aftershave, lemon, organic fertiliser, cinnamon, wet earth, burning (guiacol), and Texta (butanol)-weak, -medium, and -strong. The participants first tried to identify the odorant and then reported their visual colour association (the best-matching colour). Next they rated the irritancy, familiarity, and their hedonic liking for each of the odorants on 7-point scales. During the experimental procedure, the participants were only exposed to olfactory and visual stimuli, picking a specific colour from a standard colour palette. Somatosensory, auditory, and gustatory associations were subsequently investigated by means of open-ended questioning. The procedure was repeated 2 weeks later with the odorants presented in a different order. Colour brightness correlated with the perceptual attributes of the odorants. Odours rated as irritating, intense, and unpleasant were associated with brighter colours, thus stressing the importance of hedonics to the crossmodal matching in this case. More nameable/familiar (i.e. identifiable) odour sources were associated with more saturated colours, thus hinting at a role for semantics.

Kim (2013) presented Korean participants with eight commercial fragrances for men and women (Channel No. 5, Opium, Shalimar, De Delicious, Aqua Pour Homme, Givenchy Gentleman, Mitsouko, and Omnia Amethyste). The participants had to pick the best-three matching colours from 120. The matching was made after 2 min in order to give the participants time to experience the perfumes’ middle notes. Immediately after picking the three colours, they then rated how similar the perfume was to 33 colours. Once again, the fragrances were non-randomly (i.e. consistently) matched to the colours. According to Kim (2013, p. 1390), the crossmodal correspondences between the fragrance families and the colours chosen were influenced by the hue (warm—cool-colour images) and tone (especially, lightness property) dimensions.

Maric and Jacquot (2013) presented their French participants with 16 odorants (14 equi-intense) including caramel, cucumber, lavender, lemon, lime, mint chlorophyll, mirabelle plum (at low and high intensity), orange blossom, peppermint, rose, pineapple, shallot, smoked, violet, and wild strawberry. Five of these odorants were similar to those tested by Demattè et al. (2006). Two pairs of similar odorants (namely lemon/lime and peppermint/mint chlorophyll) were included in the hope of teasing out the finer nuances of odour-colour matching. The participants had to pick one of 24 colour patches varying in terms of their hue, saturation, and brightness for each odour. They also rated the odorants on 11-point scales in terms of their intensity, pleasantness, familiarity, and edibility. Significantly non-random colour matches (with two to six of the colours) were reported for all of the odorants. Of interest, subsequent data analysis highlighted a significant positive relationship between the rated pleasantness of the odorants and both the lightness and saturation (chroma) of the colour chosen, with more pleasant odours matched to colours with higher lightness and saturation.

Levitan et al. (2014) tested 14 odours associated with familiar objects (i.e. foods: burnt, candy, fish, flower, fruity, hazelnut, meat, musty, plastic, rice, soap, vegetable, vinegar, and woody) in six groups of participants including Dutch, Chinese residing in The Netherlands, Malay, German, Malaysian-Chinese, and American. The participants picked the best three and the worst three matching colours. The results revealed some common colour associations for certain of the odorants. For instance, the fruity odour tended to be associated with pink and red colours, while the musty odour was more likely to be associated with browns and oranges. A number of culture-specific differences were, though, also revealed by representational dissimilarity analysis. The greatest similarity in odour-colour matching was reported between participants from Germany and the US. Importantly, significant non-random colour choices were documented for all odours in all six of the cultural groups tested.

Jacquot et al. (2016) used the same set of odours and colours as Maric and Jacquot (2013); and Maric, Barbar, & Jacquot, 2012), and had the participants (from France and the UK) pick a colour to match each of the odours. All 16 of the odorants gave rise to a significantly non-random pattern of colour matching with the same colour associations being reported in both groups for 13 of them. Subtle differences being reported for the remaining three, namely: caramel, mint chlorophyll, and peppermint.

Gilbert et al. (2016) had their participants pick colours to match emotions and a variety of consumption scenarios, including the following ‘I smell hot spiced cider’. As one might have expected, the majority of the participants’ responses in this case were in the golden-brown colour range, suggesting that they were indeed matching to the imagined colour of the source object (cider). However, given that the other situations tested by Gilbert et al. involved imagining tasting something, the colour matches are a little harder to unequivocally attribute to orthonasal olfaction rather than, say, retronasal olfaction or taste/flavour, and hence are not discussed further here.

Nehmé et al. (2016) conducted a three-country study involving participants from France, Taiwan, and Lebanon who picked a colour from the 24 choices offered for each odour. A reasonably high degree of agreement was reported in terms of the broad colour palette chosen for specific fragrances along with a degree of culture specificity. As demonstrated elsewhere, food and floral odours were consistently matched to colours. One striking cross-cultural difference was that pineapple odour was reliably matched with yellow by Taiwanese participants, while being matched with red by many of the French participants. (It is perhaps worth noting that one of the dominant odour molecules in both strawberries and pineapple is furaneol; pine berries, otherwise known as white strawberries, are also described as tasting of pineapple; see Zheng et al., 2012.) While the colours chosen were significantly non-random for all odours in all three groups, for 14 of the 16 odorants there were significant differences in primary colours chosen across the three cultural groups.

Adams and Doucé (2017) conducted a study with 284 students presented with 32 odours comprising a range of object-related odours and fragrances. For each odorant, the participants had to complete 19 semantic differential scales anchored with pairs of words, that included bright-dim and light-dark scales that can, perhaps, be considered written equivalents of von Hornbostel’s (1931) perceptual grey scale. The odorants were assigned significantly different values on these line scales. So, for example, the brightest and lightest odorants were lemon, apple, and peach, whereas the dimmest and darkest odorants were coffee, cinnamon, and chocolate (making one wonder whether these mappings may actually have been driven by the colour of the source objects). As has been documented previously, robust shape associations were also documented in response to the odorants.

De Valk et al. (2017) tested odour-colour associations in three linguistically diverse groups, the Maniq (a hunter-gatherer group), Thai, and Dutch participants. The participants were presented with 15 concrete food odorants designed to be familiar to either the Thais, the Dutch, or both, including: fermented petai beans, dried durian, shrimp paste, coconut milk, galangal, mustard, licorice, red wine, peanut butter, cheese, banana, tobacco, garlic, canned fish, and cooked rice. The participants picked one of 84 colour chips varying systematically in perceptual steps in terms of hue, value, and chroma. The participants completed the task twice separated by an average interval of 2 h, and then tried to name the odours. Intriguingly, the Maniq use abstract terms to describe the odours. So, for example, de Valk et al. (2017, p. 1172) describe how another hunter-gatherer group with similar naming tendencies labelled the odours as follows: *‘In Jahai, for example, ltp t describes fragrant smells coming from various flowers, perfumes, and bearcats, whereas pl eŋ describes smells coming from, amongst other things, blood, raw fish, and raw meat.’* The rate of consistent colour-odour matches was just 1.5% amongst the Maniq, as compared to 18.5% consistency for the other two groups. According to De Valk et al. (2017, p. 8), these findings suggest that one of the key strategies underpinning the association of colours with odours is via language.

Goubet et al. (2018) reported a study in which they assessed colour-odour associations cross-culturally in French and North American participants. Additionally, they compared 6–10-year-old children (*N* = 386) with adults (*N* = 137). Eight odorants were chosen to be familiar to one cultural group and/or the other: lavender, grenadine syrup, methyl salicylate (found in root beer and wintergreen), maple syrup, strawberry, butyric acid, bergamot, and chamomile. The participants had to pick one of eight colour patches (orange, red, yellow, white, purple, green, brown, and blue) for each smell stimulus. Thereafter, they gave hedonic ratings for each odour, followed by familiarity ratings. Finally, the participants tried to identify the odorants. By 6 years of age, some culture-specific colour associations were already evident for certain of the odorants. Note here that infants of only a few months of age have been shown to internalise arbitrary correspondences between cup colour and the taste of the solutions contained within (Reardon & Bushnell, 1988). There was some evidence that the associations, or crossmodal correspondences, changed with age, and also that the participants’ familiarity with the odorants, as well as correct/near-miss identification, tended to increase with age. Taken together, these results therefore suggest that children and adults formed both shared and culture-specific odour-colour associations. Subsequent data analysis revealed that, in contrast to early studies that have typically used more abstract olfactory stimuli (i.e. those odorants without an obvious source object), odour hedonics explained only about 16% of the colour responses chosen.

Kaeppler (2018) had 30 participants visualise their colour associations with odour using a drawing tablet. The participants were free to pick the colour and shape that best matched each of the ten odorants, namely: anise, cinnamon, coconut, elder, isoamyl acetate, lemon, mustard, patchouli, peppermint, and ethyl acetate, chosen on the basis that naming the source would be easy, hard, or intermediate. Though the participants were allowed to respond freely, their responses were categorised into one of six hue categories (red, yellow, green, cyan, blue, and magenta). Significantly non-random colour associations were observed for seven of the ten odorants. So, for example, lemon was associated with yellow. More consistent colour associations were reported for those odorants where the participants found it easier to correctly identify the source. What is more, divergent colour choices could often be traced back to divergent source attributions. Of note, there was little evidence for odour pleasantness or intensity ratings modulating the participants’ colour choices. Thus, once again, these results support a semantic account of at least some colour-odour correspondences.

### Interim summary

One important factor determining the kind of colour match obtained that has emerged from the literature relates to the familiarity and identifiability of the odorants studied. While many studies have chosen to use familiar and readily identifiable odours (such as lemon, strawberry, spearmint, caramel, and lavender), the use of such odorants is likely to give rise to semantic (source object based) mappings. Perhaps unsurprisingly, it would seem that whether or not the odour can be linked to a particular object is likely key to whether participants make a more semantic or perceptual match. While much of the research would implicitly seem to have been built on the association (i.e. statistical co-occurrence) account of such crossmodal correspondences, emotional-mediation has also been shown to play a part, at least for those odours (such as perfumes) that are unfamiliar and hence cannot necessarily be linked to the other modality-specific properties of any particular source object or stimulus (e.g. Schifferstein & Tanudjaja, 2004).

Given that a number of the studies that have just been mentioned went some way toward matching the intensity and, in some cases, also the pleasantness, of the odorants involved, it should perhaps not come as a surprise that these aspects of participants’ response to the odours were found to have little impact on the pattern of colour choices obtained. However, in the few studies where the intensity of the odorants has deliberately been varied, a lightness-odour intensity association has been documented (e.g. Kemp & Gilbert, 1997; von Hornbostel, 1931). Von Hornbostel (1927/1950, p. 210) notes that until as late as the period of Middle High German the word *bright* was used to describe a sound. To the extent that this linguistic/semantic descriptor is used for stimuli in every sensory modality, it might provide support for the semantic/linguistic account of at least certain odour-colour crossmodal correspondences. In particular, a crossmodal dimensional correspondence has been observed between increasing colour lightness and less intense olfactory stimuli. That said, it remains rather unclear from the evidence published to date whether any such crossmodal match reflects a direct link between the sensory inputs in terms of their intensity (or brightness, as perhaps von Hornbostel would have imagined) or rather merely the result of separate relative judgments along modality-specific intensity scales. The latter being the suggestion made by Cohen (1934.; see also Hartshorne, 1934; Ryan, 1940) Here, one might consider whether Börnstein’s (1936) notion of sensory brightness equivalence across the modalities might be the dimension along which stimuli correspond. It was Von Schiller’s (1933) interest in the notion of sensory brightness that motivated him to demonstrate that, given the choice, fish tended to swim toward the corresponding odour following training to respond to one of a pair of visual brightnesses. No such behavioural bias was observed in response to olfactory stimuli without such visual training.

In terms of the likely neural underpinnings of odour-colour crossmodal correspondences, research by Österbauer, Matthews, et al. (2005), mentioned earlier, highlighted greater activation in the orbitofrontal cortex (OFC) and insular cortex in response to congruent (and hence hedonically more pleasant) colour-odour pairs (e.g. spearmint-turquoise and strawberry-red) than for incongruent combinations of stimuli (see also Hörberg et al., 2020). Both unisensory and multisensory combinations of olfactory and visual stimuli were presented, including lemon (yellow), spearmint (turquoise), caramel (brown), and strawberry (red). It is worth noting that these odorants were all associated with edible source objects and would likely have been very familiar to the participants. It might presumably be expected that distinct neural substrates would be engaged were unfamiliar (i.e. inedible) odorants to have been used, and/or should the correspondence be based on emotional mediation (Schifferstein & Tanudjaja, 2004), or stimulus intensity (Kemp & Gilbert, 1997; von Hornbostel, 1931), rather than based on the congruency of familiar pairings of colour and odour.

## Commercialising odour-colour correspondences: colour-based scent expectations

Having demonstrated the existence of a number of consistent cross-cultural colour-odour correspondences, the next question to ask, especially for a journal such as this, is how the findings can and, in some cases, are already being applied. These days the implications of such research would appear to have been realised primarily in the fields of marketing and design rather than amongst artists as was once briefly the case around the end of the nineteenth century. The majority of perfume brands are already closely linked to specific colours, or colour schemes (see https://www.fragrantica.com/colors/ to search through commercial perfumes by the dominant colour of the bottle). Just think, for example, of *Blue* or *Red* by Hugo Boss. There is, then, a sense in which many perfume brands may already intuitively base their visual designs on the crossmodally corresponding colour palette (Kim, 2008).

Consistent with such a view, Kim (2008) analysed the colour palate of 200 commercial fragrance bottles and their packaging and demonstrated that a fragrance family tends to be reflected in the colour palette chosen. Kim then took three commercial perfumes and presented the fragrances blind to participants who had to pick the matching colour. Even though the participants reported being unfamiliar with the fragrances, they nevertheless generally picked colours that were in agreement with the brand colours; thus, suggesting that the colour schemes of commercial fragrances (or at least the successful ones) may well already be built on colour-odour crossmodal mappings.

At the same time, however, one might wonder if it matters whether fragrance and colour scheme correspond crossmodally. In the case of a new fragrance, could customers actually be taught to associate it with pretty much any colour scheme? How easy is it, in other words, to teach people a new association between a novel fragrance, and an arbitrary colour, or colour scheme? While the relevant research has yet to be conducted, the emerging body of sound symbolism research (which has been linked to the crossmodal correspondences; see Spence, 2011) suggests that natural correspondences tend to facilitate language learning (e.g. Imai et al., 2015; Imai & Kita, 2014; Imai, Kita, Nagumo, & Okada, 2008). Hence, one might expect the same to be true in the case of customers acquiring, or internalising, novel colour mappings for new fragrances. It would be good to see more learning experiments of this sort conducted/published in the future.

Optimising colour-odour congruency likely makes sense from the point of view of processing fluency (e.g. Barkat, Thomas-Danguin, Bensafi, Rouby, & Sicard, 2003; Kim, 2008, 2013; Lunardo & Livat, 2016; Schifferstein & Howell, 2015; see also Porcherot, Delplanque, Gaudreau, & Cayeux, 2013; Scharf & Volkmer, 2000; Togawa, Park, Ishii, & Deng, 2019). Note that this is not only achieved by the use of colour but also by means of the brand name and olfactory imagery/written description on the packaging and any relevant advertising (Meng, Zamudio, & Jewell, 2020). Incongruent combinations of colour and fragrance could presumably also be used, though this tends to be a much more challenging strategy to pull off successfully in the marketplace (see Velasco et al., 2016).

**Fig. 1:**
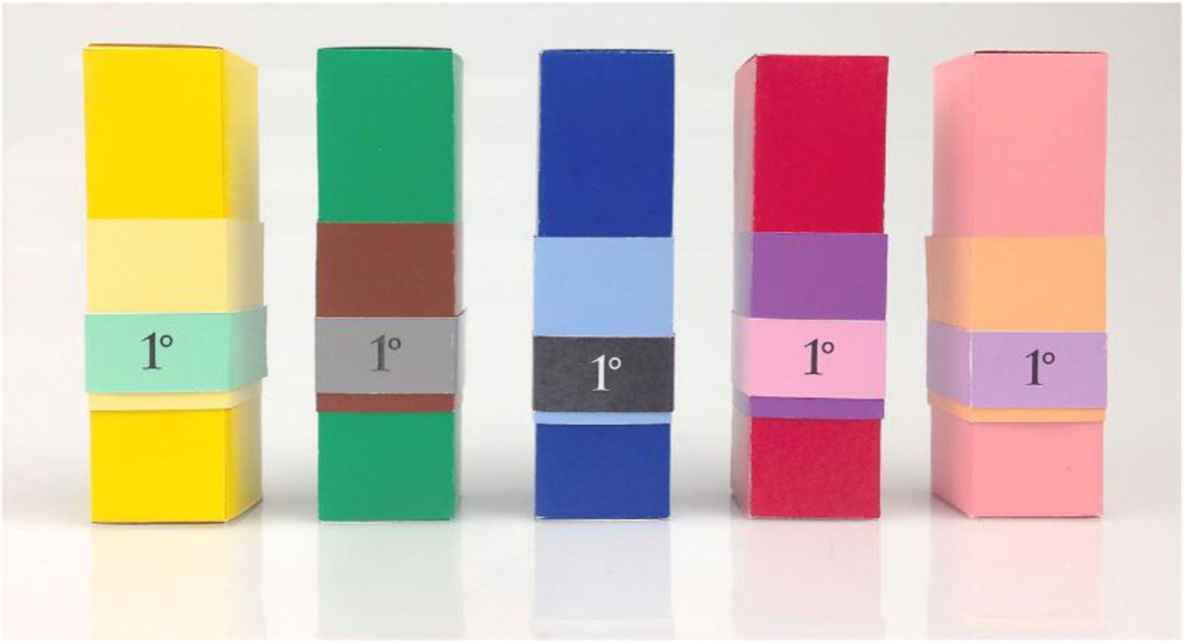
The five prototype packages designed (on the basis of colour-odour crossmodal correspondences) to match the five commercial fragrances in Schifferstein and Howell’s (2015) study. From left-to-right, packaging schemes designed to match DKNY, Miss Dior, Kouros, Wish, and Paris, respectively. (see online version for colour image

But just how strong is the crossmodal correspondence between colour and fragrance when it comes to the colour scheme used in product packaging? In one intriguing study, Schifferstein and Howell (2015) had 66 Dutch female participants rate the appropriateness of five exemplars of product packaging that incorporated three corresponding colours for five commercial fragrances (Kouros, DKNY, Wish, Paris, and Miss Dior; see Fig. 1). The results revealed some significant differences in terms of the appropriateness of the colour scheme to the scent for four out of five of the fragrances, though, that said, variation in appropriateness (goodness-of-fit ratings) between the best and worst colour-odour pairings was always less than 3 on a 9-point scale. Schifferstein and Howell subsequently obtained broadly similar appropriateness ratings in a sample of 80 North American students; thus, hinting at a degree of cross-cultural generality to their findings.

**Fig. 2:**
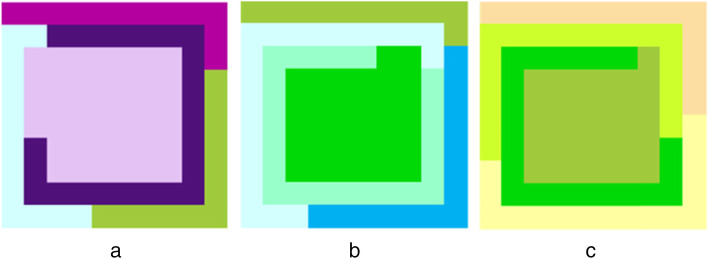
Chromatic cards® generated for the study by Jacquot et al. (2016), each containing five distinct shades of colour: **a** lavender, **b** peppermint, and **c** cucumber; (see online version for colour image)

A patented scientific tool based on a neural network algorithm for colour forecasting has also been developed at Lorraine University, France (patent FR no. 1255688). This tool generates chromatic cards that (or so it is claimed) can represent any odour on the basis of its chemical composition and a library of sensory descriptions. For instance, in the latter part of a study by Jacquot et al. (2016, discussed earlier), three chromatic cards were created to convey the smell of lavender, cucumber, and peppermint (see Fig. 2). French and British participants (119 in total) were shown the cards and had to describe the odours they associated with it. Thereafter, they were forced to choose which card they would associate with the three named odours. The results showed that the chromatic cards evoked the appropriate odour at a level that was higher than would have been expected for the best of the individual colours. In terms of the forced choice discrimination task, the participants from both the UK and France matched the correct chromatic card to each of the three odorants in 60–70% of trials. Such results therefore hint at the promise that combining corresponding colours might have in terms of linking to specific odorants.

Heatherly et al. (2019) recently presented 50 North American participants with five odorants relevant to a chardonnay white wine, namely buttery, citrus, floral, smoky, and vegetable-forward (bell pepper). In an initial projective mapping study, the participants had to match each odorant (sniffed in the headspace over a glass of chardonnay wine to which the odorant had been added) with one of four colours (yellow, red, green, and brown). The participants also had to match the wine bouquets to one of four shapes varying in terms of their angularity (round versus angular) and complexity (simple versus complex). The chardonnay odours were mostly matched with yellow and green. The results also revealed that the vegetable-forward wine aroma was matched with the angular shape though no colour preference was observed in the case of this particular olfactory note.

**Fig. 3:**
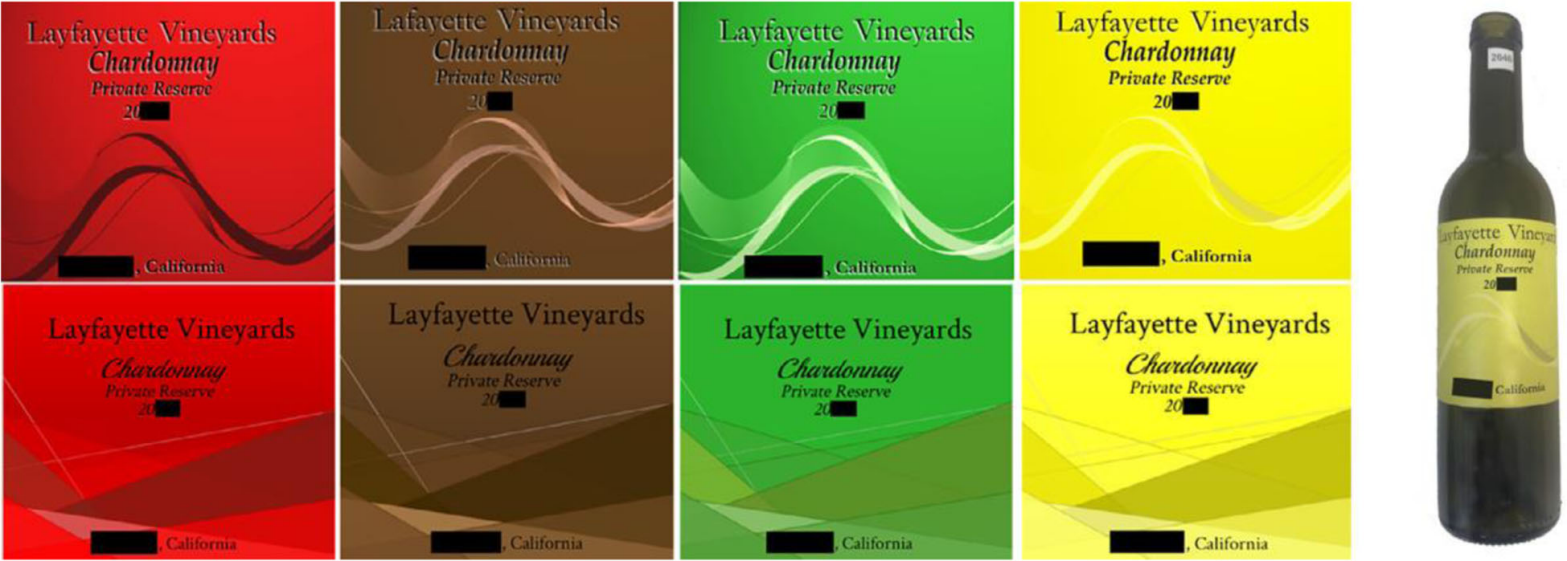
Wine labels affixed to wine bottles (comprising 2 levels of angularity – rounded and sharp) as well as four colours (red, brown, yellow, and green). Heatherly et al. (2019) assessed the orthonasal olfactory expectations set by these wine labels, (see online version for colour image)

In a second experiment, eight wine labels (varying in terms of their angularity and background colour; see Fig. 3) were created (see Matthews, Simmonds, & Spence, 2019, for a similar approach to the use of colour and shape cues in the design of congruent packaging in other product categories; see also Hoffman & Ralph, 2013). In this case, the participants rated how well the odour in the glass matched each of the wine labels (displayed on a bottle, shown on the right of the figure) on a 10-point scale. The yellow labels constituted a better match for all the chardonnay wine odours, except the vegetable-forward wine, which was matched equally with all four label colours. These results therefore suggest that chardonnay wine may be the most relevant semantic level at which people base their crossmodal colour associations, rather than colour associations being driven by the specific component odours (e.g. vegetative – green for green bell pepper, or smoky – brown). This conclusion is consistent with the earlier suggestion by Zellner et al. (2008) that it was a fragrance being considered, or labelled, as male or female (rather than the specific olfactory notes) that determined the dominant colour palette (i.e. blue or pink) participants chose to match to the unisex fragrances.

In summary, while there is undoubtedly growing interest in the commercial application of the findings of odour-colour crossmodal correspondences research, it should be noted that much of it is built on the assumption that insights from associations that have been documented in one direction will necessarily make useful predictions about the existence of associations in the reverse direction. However, the majority of cognitive research studies reported in the previous section had their participants associate colours with odours, rather than associating odours with colours. In the marketplace, it is the colour of the product and/or packaging that normally sets expectations concerning the likely properties of the contents, rather than vice versa (see Piqueras-Fiszman & Spence, 2015, for a review). And, while the bidirectionality of at least some crossmodal correspondences has been asserted (Deroy & Spence, 2013), the extent to which bi-directionality holds in the specific case of odour-colour crossmodal correspondences has yet to be established (see Cohen, 1934; Gilbert et al., 2016).

What may be especially relevant here is the difficulty that people experience in identifying odours in the absence of any other contextual cues. This, remember, is partly what makes the matching of colours to odours so intriguing in the first place. By contrast, people typically have far less difficulty labelling/identifying colours, though, at the same time, colour patches are not always linked to specific source objects in the way that olfactory stimuli so often are. It is also worth noting here that one of the key differences thought to distinguish synaesthesia from the crossmodal correspondences is the unidirectionality of the inducer-concurrent relation in the case of synaesthesia (Speed & Majid, 2018), especially in the case of crossmodal synaesthetic relations. By contrast, a bidirectional mapping is often implicitly assumed by those who have chosen to investigate crossmodal correspondences. When considering the bi-directionality question in the case of olfactory-visual crossmodal correspondences, it may be relevant to consider the very great differences in terms of the numbers of distinct receptor types for colour vision versus olfaction (i.e. three cone types versus several hundred olfactory receptor types (see Shepherd, 2006, 2012).

Looking to the future, it is easy to imagine how colour palettes may well be needed and, what is more, may help to convey (especially) complex multi-element fragrances more effectively than any single colour possibly can (see Lipps, 2018, p. 118, for one example of how Malin + Goetz use different colours to indicate the various notes in their fragranced products), while all the while acknowledging the challenge of coming up with an adequate definition of perceived complexity as far as the chemical senses are concerned (see Spence & Wang, 2018, for a review). Such colour cues can then likely be made more evocative still if combined with shape cues in terms of either graphic design or in the design of typeface characteristics (see Velasco & Spence, 2019, for a review). Here, it is worth noting that various studies have already started to highlight the crossmodal correspondences that exist between odours and various shape properties (e.g. Adams & Doucé, 2017; Blazhenkova & Kumar, 2018; Crisinel, Jacquier, Deroy, & Spence, 2013; Hanson-Vaux, Crisinel, & Spence, 2013; Kaeppler, 2018; Ngo et al., 2013; Seo et al., 2010). Hence, there is already a growing body of academic research for designers to work with.

### Problems with applying basic colour-odour crossmodal correspondences research

Ultimately, though, one of the key problems with applying the findings of colour-odour crossmodal correspondences research may be that the cross-modal mapping is generally too coarse to be useful. What this means is that, by itself, a single colour on product packaging, say, is unlikely to be sufficiently specific in terms of its meaning or olfactory association (unless, perhaps, that particular hue has become closely linked with a specific brand). In fact, the meaning of colour is often constrained by the use of visual imagery and text in product packaging. Once multiple colours are combined, though, an additional factor suddenly comes into play, namely the harmony between the colours themselves; this might, in turn, come to interfere with any crossmodal mapping between the individual colours and odours (see Schifferstein & Howell, 2015; cf. Favre & November, 1979; Woods, Marmolejo-Ramos, Velasco, & Spence, 2016; Woods & Spence, 2016). Potentially relevant here, Langner (1997) found that abstract images containing two or three colour fields appeared to be less effective at triggering odour associations than those images with fewer colours.

Outside of the context of colour-odour correspondences research, it is worth noting that colours are rarely presented/seen in isolation. Given that combinations of colours are typically used in design practice, the question of the relative strength of intramodal versus crossmodal perceptual grouping suddenly comes to the fore. It would intuitively feel as though the intramodal grouping of colours dominates over, or precedes, the crossmodal grouping of colours with odours (see Spence, 2015b, for a review). This, then, perhaps makes sense of Kandinsky’s decision a century ago to focus on the relation between colour and visual form rather than crossmodal relations (Dreksler & Spence, 2019; Kandinsky, 1977; see also Kimura, Wada, & Noguchi, 2005). Indeed, a number of intramodal questions arise just as soon as the decision is made to present multiple colours – both the question of foreground/background colour relations (cf. Woods et al., 2016), but also the question of colour harmony (Ou, 2015; Shen, Yuan, Hsu, & Chen, 2000; see also Albers, 1963). Put simply, even though two or three colours, when presented individually, might correspond well with a given fragrance, that provides no guarantee that combining those colours will necessarily also give rise to a good match for the odour. Hence, in the future, marketing-related colour-odour correspondence research will probably need to assess the crossmodal odour mappings that are elicited by different combinations of colours, rather than try to infer the appropriate mappings based on the specific individual colours that people happen to associate with odours.

One other important consideration as far as designing colour schemes to correspond to commercial perfumes is concerned is that the latter typically evolve over time, with top, middle, and base notes predictably revealing themselves one after the other (Kim, 2008). This might suggest that a temporally evolving colour palette (and/or a transition from light to dark colours) would really be needed if one wanted to fully capture the various stages in the evolution of a particular fragrance. That is, different colours would likely be associated with the top, middle, and base notes of commercial fragrances (Stamelman, 2006). That said, an argument can perhaps also be made that it is likely to be the top notes that help to create the first impression that may be most important, and that perhaps there is, anyway, less variation in terms of base notes (see Deroy, Crisinel, & Spence, 2013).

## Conclusions

The last decade or so has seen a remarkable expansion of research interest in the crossmodal correspondences (see Spence, 2011, 2018, for reviews). Building on early research by Marks (Marks, 1978, Marks, 2004), that focussed primarily on audiovisual correspondences (see Sathian & Ramachandran, 2020, for recent reviews and discussion), there has undoubtedly been a widening of research interest in the existence/nature of the crossmodal correspondences that exist between a range of different sensory modalities and stimulus dimensions. There has, for instance been much interest in the correspondences between thermal cues and colours (see Spence, 2020b, for a review) through associations between music and colour/paintings (see Spence, 2020a, for a review), and even colours and basic tastes (see Spence, 2019b; Spence et al., 2015, for reviews). Colour-odour correspondences can thus be seen as but one strand of this broadening academic interest in the correspondences that, perhaps unusually, has engaged the interests of both basic researchers and those working in various applied domains (e.g. Déribéré, 1971, 1973a, b; Guillot & Guillot-Allegre, 1953; Langner, 1997; Popova, 2004).

And yet, as made clear in the ‘Introduction’ to this review, there is, in fact, a long history of interest, though often wrapped up, at least initially, in the separate literature on what is often referred to (rightly or wrongly) as synaesthesia (e.g. Azari, 1924; Baudelaire, 1857, 1954; see also Kandinsky, 1977). A number of artists and composers (in the opening decades of the twentieth Century), from Cèzanne to Scriabin (Hull, 1927; Runciman, 1915), through to little-known Futurist artists such as Azari (1924) and Carrà (1973) were undoubtedly curious about the connections, or correspondences that linked colours with odours (see Verbeek, 2017, for a review). However, one of the stumbling blocks that stymied this early interest was that the phenomenon was primarily considered in terms of ‘synaesthesia’, what the Futurists were called ‘*syn-olfactismo*’ (Marinetti, 2014; see also Fleischer, 2007; Shepherd-Barr, 1999).

Basing one’s artistic design decisions on the definitionally idiosyncratic associations between sensory impressions (see Grossenbacher & Lovelace, 2001, for one popular definition of synaesthesia that incorporates idiosyncrasy) would, a priori, seem unlikely to deliver the widely comprehensible connections that the artists were striving for when trying to communicate, or translate, sensory impressions, for their prospective audiences (cf. Runciman, 1915). It can be argued that a similar problem faces the field of synaesthetic design that has started to become popular in recent years (e.g. Haverkamp, 2014; Merter, 2017; Spence, 2012), at least if taken literally as design solutions that are based on the unusual correspondences (or inducer-concurrent pairings) experienced by synaesthetes (see Spence, 2015c).

Given that recent empirical research has increasingly started to distinguish between synaesthetic and crossmodal correspondences (e.g. Deroy & Spence, 2013), the establishment of consistent mappings between colour (and shape, etc.) and odour; associations that, in some cases at least, work across both culture and age (Goubet et al., 2018), would perhaps appear to provide the necessary information for those wanting to translate between this particular pair of senses, while at the same time highlighting those colour-odour correspondences where cultural/experiential differences mean that any such universal translation is unlikely to succeed, or will need further constraining by the use of text or imagery (Meng et al., 2020). Given the evidence that has been published to date, such cross-sensory translation would appear most likely to be effective under those conditions in which the odour-colour mapping is universal – this might either be because people’s experience is likely to be shared in terms of the mapping, or else because the olfactory stimulus itself is entirely unfamiliar and hence the correspondence is more likely to tap into emotional mediation or structural (i.e. intensity-based) account.

Though, once again, it is worth stressing that just because a particular odorant may itself be unfamiliar, that provides no guarantee that the person smelling it might not be reminded of a familiar odorant that itself has typically been experienced together with a particular colour in the past. What is more, as Runciman (1915) noted long ago, the emotional associations that we bring to odours also likely differs from one individual to the next. At the same time, however, even in the best-case scenario, it should be remembered that the colours mapped to odours never show perfect agreement. And even for certain odours that are strongly and unequivocally mapped to colour amongst certain participants, the research sometimes shows very different, yet seemingly equally consistent, mappings with different colours in different cultures. Furthermore, people often say that they find the task challenging while, at the same time, rating it as an intuitive thing to do.

Nevertheless, there is growing commercial interest in capitalising on the correspondences in order to more effectively convey the olfactory/flavour attributes of products by means of the use of colour, shape, and other cues (e.g. Adams & Doucé, 2017; de Sousa, Carvalho, & Pereira, in press; Heatherly et al., 2019; Lick, König, Kpossa, & Buller, 2017; Lunardo & Livat, 2016; Sugrue & Dando, 2018). However, as we have just seen, as soon as colours and forms are combined in design practice, the focus of an observer’s attention may well switch to one of intramodal perceptual grouping/evaluation (see Spence, 2015b). Such intramodal judgments of harmony are, then, in danger of dominating over crossmodal judgments of correspondence.

In closing, it is tempting to ask how the strength/robustness of the crossmodal correspondences that have been documented between odour and colour compare to those established between colour and taste (Spence, 2019a, b; Spence et al., 2015) or between odour and auditory qualities such as pitch and timbre (Deroy et al., 2013). As yet, there is simply no standardised means of assessing the relative strength or robustness of different types of crossmodal correspondence. However, according to the statistical account, one might consider that a more robust binding, or coupling, prior would be internalised when the component stimuli regularly co-occur. Here, one might also consider the question of how easy it is to update, or modify, existing crossmodal correspondences when exposed to a new mapping in the environment. However, the answer to this question may ultimately be complicated by the question of whether one is talking about statistically, structurally, semantically/lexically, or emotionally mediated correspondences. There are, in other words, still a number of key questions, both theoretical and practical, to be addressed in order to make the most of the ‘coloured scents’ that first captured the interest of artists a little over a century ago. As has hopefully been made clear by this review, progress in this area of research is likely to be facilitated by an approach that combines theoretical insights concerning the crossmodal correspondences (and how they differ from synaesthesia), together with a consideration of their likely practical implications for the worlds of design and marketing.

## Data Availability

Not applicable
